# The Poly-Glutamate Motif of GmMATE4 Regulates Its Isoflavone Transport Activity

**DOI:** 10.3390/membranes12020206

**Published:** 2022-02-10

**Authors:** Yee-Shan Ku, Sau-Shan Cheng, Ming-Yan Cheung, Yongchao Niu, Ailin Liu, Gyuhwa Chung, Hon-Ming Lam

**Affiliations:** 1 School of Life Sciences and Centre for Soybean Research of the State Key Laboratory of Agrobiotechnology, The Chinese University of Hong Kong, Hong Kong, China; chengsaushan@yahoo.com (S.-S.C.); cheungmy@cuhk.edu.hk (M.-Y.C.); niuyongchao@link.cuhk.edu.hk (Y.N.); merestarry@gmail.com (A.L.); 2 Department of Biotechnology, Chonnam National University, Yeosu 59626, Korea; chung@chonnam.ac.kr

**Keywords:** multidrug and toxic compound extrusion (MATE) transporter, proton motive force, proton gradient, poly-glutamate motif, acidic amino acid, isoflavone, legume, soybean

## Abstract

Multidrug and toxic compound extrusion (MATE) transporters in eukaryotes have been characterized to be antiporters that mediate the transport of substrates in exchange for protons. In plants, alkaloids, phytohormones, ion chelators, and flavonoids have been reported to be the substrates of MATE transporters. Structural analyses have been conducted to dissect the functional significance of various motifs of MATE proteins. However, an understanding of the functions of the N- and C-termini has been inadequate. Here, by performing phylogenetic analyses and protein sequence alignment of 14 representative plant species, we identified a distinctive N-terminal poly-glutamate motif among a cluster of MATE proteins in soybean. Amongst them, GmMATE4 has the most consecutive glutamate residues at the N-terminus. A subcellular localization study showed that GmMATE4 was localized at the vacuolar membrane-like structure. Protein charge prediction showed that the mutation of the glutamate residues to alanine would reduce the negative charge at the N-terminus. Using yeast as the model, we showed that GmMATE4 mediated the transport of daidzein, genistein, glycitein, and glycitin. In addition, the glutamate-to-alanine mutation reduced the isoflavone transport capacity of GmMATE4. Altogether, we demonstrated GmMATE4 as an isoflavone transporter and the functional significance of the N-terminal poly-glutamate motif of GmMATE4 for regulating the isoflavone transport activity.

## 1. Introduction

Multidrug and toxic compound extrusion (MATE) transporters are a family of active membrane transporters generally consisting of 12 transmembrane domains (TMDs) [1]. The 1st to the 6th TMDs are usually termed as the N-lobe while the 7th to the 12th TMDs are usually termed as the C-lobe [2,3]. MATE transporters have been reported to be localized at membranes including plasma, vacuolar, and mitochondrial membranes, the chloroplast envelope, and the surface of small vesicles [1]. In prokaryotes, MATE transporters transport the substrate across the membrane in exchange for Na^+^ or H^+^, while in eukaryotes, they tend to transport the substrate in exchange for H^+^ only [4]. The electrochemical gradient across the membrane drives the transport of the substrate. Structurally, MATE proteins are generally characterized by the typical 12 TMDs [5]. However, the different amino acid residues of different MATE proteins enable different substrate specificities [1,6,7]. In plants, MATE transporters have been reported to be responsible for the movement of alkaloids, phytohormones, ion chelators, and flavonoids including anthocyanins and isoflavones across membranes [1,6,7,8,9]. The transport direction is largely associated with the nature of the substrate to be transported. For example, it has been reported that GmMATE1 and GmMATE2 mediate the transport of isoflavones into the vacuole for storage [7]. In plant genomes, *MATE* genes usually compose a big family. For example, in studies reporting genome-wide identification of *MATE* genes, 117, 67, 53, 56, and 40 *MATE* genes were identified in *Glycine max*, [10], *Solanum lycopersicum* [11], *Oryza sativa* [12], *Arabidopsis thaliana* [13], and *Medicago truncatula* [9], respectively. The need to transport various metabolites by MATE proteins with different substrate specificities may be the possible reason behind the big MATE families in plants.

Research has been done to understand the protein structure of MATE transporters and the roles of the various domains within that structure. For example, it was found that the protonation of acidic amino acid residues at the C-lobe of the transmembrane domain of AtDTX14 (*Arabidopsis thaliana* detoxification efflux carrier 14) resulted in a structural change in the transmembrane helix 7, and thus, regulated the transport mechanism [2]. It was also suggested that the binding of protons at these acidic residues played a role in the coupling of the proton-motive force to regulate the substrate transport [2]. Previous studies also suggested the importance of the N-terminal domain (NTD) in substrate and ion binding [3,14]. Based on the crystal structure of the MATE protein from *Bacillus halodurans* (DinF-BH), it has been reported that D40 in TMD1 was the key cation-binding residue, which also bound to the substrate to be transported [3]. The D40N mutation led to drastically reduced binding of the drug to be transported, and the ability of the MATE protein to release H^+^ upon drug binding was abolished [3].

Most research has focused on studying the structural importance of the TMDs. However, the non-transmembrane domains of these transporters, i.e., the N-terminal tail and the C-terminal tail, also play significant regulatory roles. It has been reviewed that the N- and the C-terminal tails of transporters are involved in other aspects of the regulation of the transporter proteins, such as the sorting to organelle, ubiquitination and turnover, and transport activity and substrate specificity [15]. Several transporters have been reported to have conserved N- ad C-termini among a group of organisms [15]. For example, nucleobase-ascorbic transporters (NATs) have a nearly absolutely conserved sequence in the N-terminus among fungi [15]. This conserved motif is not found in other organisms such as prokaryotes, slime molds, protists, plants, or metazoans [15,16]. Another example is the C-terminus of the yeast amino acid polyamine-organocation (APC) superfamily. It was found that the C-termini of yeast APCs were more conserved than the N-termini and were predicted to be involved in the sorting, turnover, palmitoylation, and the formation of secondary structures of the transporter proteins [15]. However, the degrees of sequence conservation and the functions of the N- and C-termini of MATE transporters in various organisms including plants are largely unknown.

Through phylogenetic analyses of MATE proteins from 14 representative plant species, including those in the Leguminosae family (*Arachis ipaensis, Cajanus cajan, Cicer arietinum*, *Lotus japonicus*, *Medicago truncatula*, *Phaseolus vulgaris*, *Vigna radiata* and *Glycine max*) and other non-Leguminosae species (*Arabidopsis thaliana*, *Gossypium hirsutum*, *Helianthus annuus*, *Oryza sativa*, *Solanum lycopersicum,* and *Solanum tuberosum*), we found clades enriched with MATE proteins from Leguminosae and a cluster consisting solely of MATE proteins from *Glycine max*. MATE proteins from this latter cluster, including GmMATE4 which was previously identified to be in the overlapping quantitative trait loci (QTLs) regulating the contents of antioxidants, phenolics, and flavonoids in soybean seeds [17], have a conserved poly-glutamate motif at the domain predicted to be inside the vacuolar compartment (vacuolar domain) of the N-terminus. Here, we demonstrated GmMATE4 as an isoflavone transporter and the role of the poly-glutamate motif in regulating the transport activity of GmMATE4.

## 2. Materials and Methods

### 2.1. Phylogenetic Analysis, Protein Sequence Alignment, Topology Prediction, and Protein Charge Prediction

The protein sequences of MATE family representatives from 14 plant species, including Leguminosae (*Arachis ipaensis*, *Cajanus cajan*, *Cicer arietinum*, *Lotus japonicus*, *Medicago truncatula*, *Phaseolus vulgaris*, *Vigna radiata,* and *Glycine max*) and non-Leguminosae (*Arabidopsis thaliana*, *Gossypium hirsutum*, *Helianthus annuus*, *Oryza sativa*, *Solanum lycopersicum,* and *Solanum tuberosum*), were retrieved from the NCBI or Phytozome database and used for gene family clustering. The longest protein sequences of each gene were retained and all-against-all BLASTP [18] was used to calculate pairwise sequence similarities with a *p*-value cutoff of 1 × 10^−5^. Then, the homologous genes were identified by OrthoMCL [19] with the use of default parameters. The MATE protein sequences were aligned by ClustalW in MEGA11 [20]. The topology prediction of MATE proteins was done with the use of Protter [21]. Then, the N-termini of the MATE proteins were subjected to charge prediction by Prot-pi [22] using the pKa values based on ProMoST [23] for calculating the isoelectric point.

### 2.2. Subcellular Localization Study

*GmMATE4*, *GmMATE4Δ3ala*, and *GmMATE4Δ7ala* were amplified from pGBKT7∆BD-*GmMATE4*, pGBKT7∆BD-*GmMATE4Δ3ala*, and pGBKT7∆BD-*GmMATE4Δ7ala* as the templates, respectively, with the use of PrimeSTAR GXL DNA Polymerase (R050B, TaKaRa). The DNA fragments were subjected to restriction digestion, and then, fused to the 5′ end of *green fluorescent protein* (*GFP*). The fusion constructs were cloned downstream of the CaMV 35S promoter of the vector V7. The primers and restriction enzymes used are listed in Supplementary Materials Table S1. Restriction enzymes and T4 DNA ligase used for cloning were from New England Biolabs.

The plasmids were, then, coated onto gold particles and bombarded into onion epidermis with the use of the Biolistic^®^ PDS-1000/He Particle Delivery System (Bio-Rad, Hercules, CA, USA), according to manufacturer’s protocol. Each 3 mg of gold particles (1.0 μm diameter, #1652263, Bio-Rad, Hercules, CA, USA) was washed with 1 mL 70% EtOH by vortexing for 5 min. After that, the gold particles were allowed to be incubated in the 70% EtOH before being fast spun for 5 s. Then, the supernatant was removed, the particles were added with 1 mL sterile water by vortexing, and then, the gold particles were allowed to be incubated in water for 1 min before being spun for 2 s. Then, the supernatant was removed. The water-wash steps were performed three times. After removing the supernatant, the washed particles were added with 50 μL sterile 50% glycerol. Then, 5 μL plasmid DNA (1 mg/mL), 50 μL CaCl_2_ (2.5 M), and 20 μL spermidine (0.1 M) were mixed with the gold particle by vortexing. After the mixing, the gold particles were allowed to be incubated in the mixture for 1 min before being fast spun for 2 s. Then, the supernatant was removed. Without being disturbed, the gold particles were washed using 140 μL 70% EtOH, followed by being washed with 140 μL absolute EtOH. After removing the supernatant, 48 μL EtOH was added to the gold particles. The gold particles were resuspended by pipetting before the bombardment. All steps were performed at room temperature. For each bombardment under 1100 psi, 0.75 mg of the gold particles coated with 1.25 µg of plasmid DNA were used. The onion epidermis was left at room temperature overnight before being observed using a confocal microscope (Olympus FV1000, excitation 488 nm, the emission signal was collected between 500 and 545 nm). The images were processed by a FV10-ASW 4.2 Viewer. The experiment was performed twice. All cells showing the green, fluorescent signal (≥11 cells) from the two experiments showed similar patterns.

### 2.3. Yeast Isoflavone Uptake Assay

The coding sequence (CDS) of *GmMATE4* (*Glyma.19G120900*) was cloned from a mixture of cDNAs from the pod and the seed of cultivated soybean (*Glycine max*) accession C08, using PrimeSTAR GXL DNA Polymerase (R050B, TaKaRa, Shiga, Japan). The poly-glutamate motif of GmMATE4 was converted from EEEEEEE to AAAEEEE (GmMATE4Δ3ala) or from EEEEEEE to AAAAAAA (GmMATE4Δ7ala) with the use of specially designed primers. Then, the DNA fragments were cloned into the vector pGBKT7∆BD [7]. The primers and restriction enzymes used are listed in Table S1. Restriction enzymes and T4 DNA ligase used for cloning were from New England Biolabs. The plasmids were transformed into the yeast strain Y2HGold (630489, Takara, Shiga, Japan) by the LiAc/PEG method [24]. To prepare competent yeast cells, overnight culture of the yeast strain Y2HGold at stationary phase, grown in YPDA (yeast peptone dextrose adenine) broth at 30 °C with shaking at 200 rpm, was inoculated to a fresh broth of YPDA at a 1:10 ratio. Then, the inoculated broth was shaken at 30 °C, 200 rpm until OD600 reached 0.4–0.6. After that, the yeast cells were pelleted at 1000× *g* for 5 min. The supernatant was removed. Then, the pelleted cells were washed by resuspending with sterile water, pelleted again at 1000× *g* for 5 min, with the supernatant removed after centrifugation. The cells were washed twice with sterile water. After that, the washed cells were resuspended in TE/LiAc buffer (0.01 M Tris-HCl, 1 mM EDTA, 0.1 M lithium acetate, pH 7.5). The yeast cells resuspended in TE/LiAc were used as competent yeast cells for transformation. Each 1 mL competent yeast cells resulted from 20 mL overnight yeast culture at stationary phase. To transform the competent yeast cells, for each construct, 0.1 μg of plasmid was mixed with 0.1 mg denatured carrier DNA (630440, TaKaRa, Shiga, Japan) and 0.1 mL competent yeast cells by vortexing. After that, the mixture of plasmid and competent yeast cells was mixed with 0.6 mL filter-sterile PEG/LiAc solution (40% PEG 4000, 0.01 M Tris-HCl, 1 mM EDTA, 0.1 M lithium acetate, pH 7.5) by vortexing. Then, the mixture was shaken at 30 °C, 200 rpm, for 30 min. After that, 70 μL DMSO was added to each transformation mixture by gentle inversion. Then, the mixture was incubated in 30 °C for 15 min, and then, on ice for 2 min. After that, the yeast cells were pelleted at room temperature, 14,000 rpm, for 5 s. After removing the supernatant, the yeast cells were resuspended in 0.1 mL sterile TE buffer (0.01 M Tris-HCl, 1 mM EDTA, 0.1 M lithium acetate, pH 7.5) and spread on 1.5% agar in synthetic dropout medium without tryptophan (SD-Trp) for selection at 30 °C. Two days after the incubation at 30 °C, the yeast colonies were picked and streaked on a fresh plate of 1.5% agar in SD-Trp to confirm the selection.

The yeast isoflavone uptake assays were performed, as described previously [7,25] with slight modifications. Untransformed Y2HGold was compared to GmMATE4-transformed yeast to rule out that the uptakes of isoflavones in GmMATE4 expressing yeast cells were solely due to the endogenous yeast proteins. Yeast cells transformed with *GmMATE4, GmMATE4Δ3ala*, and *GmMATE4Δ7ala* were used to demonstrate the functional significance of the poly-glutamate motif. Untransformed Y2HGold (wildtype) was grown in SD-Trp broth (complement with Trp) to compensate the inability to synthesize tryptophan, while the transformed yeast cells were grown in SD-Trp broth. The yeast was grown in SD-Trp broth at 30 °C with shaking at 200 rpm until OD600 of the cells reached 2.0. The cells were pelleted at 1000× *g* for 3 min. The cell pellet was washed twice with phosphate-buffered saline (PBS, pH 7.4) and resuspended in 15 mL PBS (pH 7.4). Each 1 mL aliquot of cells was treated overnight with 200 μM isoflavone or DMSO only as the mock control at 30 °C with shaking at 200 rpm. Then, the treated yeast cells were pelleted at 1000× *g* for 3 min and washed twice with 50 mM potassium phosphate buffer (pH 7.8). Intracellular metabolites in yeast cells were extracted with pure methanol by the freeze-thaw method, as previously described [7,26]. In brief, the cell pellet was mixed with 0.1 g glass powder and 500 μL methanol by vortexing. Fifty-micromolar formononetin was added as a spike. Then, the mixture was frozen in liquid nitrogen and thawed on ice. The freeze-thaw cycle was performed twice. After that, the mixture was centrifuged at 14,000× *g* for 5 min. The supernatant was filtered through an Acrodisc 13 mm mini spike 0.2 μm PVDF filter (4450, Pall Corporation, Port Washington, NY, USA). The filtered supernatant was analyzed using a Vanquish Flex UHPLC System with a UV-Diode array detector (Thermo Scientific, Waltham, MA, USA) and with the use of Acquity BEH C18 reverse-phase column (1 mm × 100 mm, 1.7 μm) (186002346, Waters Corporation, Milford, MA, USA). Three microliters of the filtered supernatant were injected into the column with the flow rate maintained at 0.1 mL min^−1^. A mixture of ultra-pure water with 0.1% formic acid (*v*/*v*) was used as solvent A while absolute acetonitrile was used as solvent B. Solvent A and solvent B at different percentages were used to make up 100% composition of the mobile phase. The sample was eluted with a gradient set at 0–5 min, 10–20% solvent B; 5–10 min, 20–90% solvent B; 10–20 min, 90% solvent B; 20–20.1 min, 90–10% solvent B; 20.1–25 min, 10% solvent B. Different isoflavone species were separated by gradient elution. Since different isoflavone species have the best solubility at different compositions of solvent A and solvent B, the elution strategy was to, first, elute isoflavones which are comparatively more soluble in aqueous solvent then other isoflavone species (0–5 min), and then, elute isoflavones by progressively increasing the percentage of solvent B (5–10 min). In the first 10 min, most of the isoflavone species are eluted (Figure S3). After that, the composition of solvent B was increased to the maximum amount to 90% to elute any molecules remaining in the stationary phase (10–20 min). The final step was to increase the composition of solvent A to 90% (i.e., 10% solvent B) to prepare the column for the next cycle of elution. The identification of compounds was achieved by comparing the retention times and UV spectra at 260 nm [27]. Vials filled with either standard solutions or samples were kept at 10 °C in the autosampler. The standards, daidzein (D-101), glycitein (GL-001), genistein (G-103), daidzin (021096), glycitin (GL-002), genistin (021050), and malonylgenistin (06–1454) were purchased from Indofine Chemical Company, Inc (Hillsborough, NJ, USA). Malonyldaidzin (PS1633–0010) and malonylglycitin (139–13831) were purchased from Chengdu Push Bio-Technology Co., Ltd (Chengdo, China) and Fujifilm Wako Pure Chemical Corporation (Chuo-Ku, Japan), respectively. The isoflavones were dissolved in DMSO and stored at −80 °C before use.

## 3. Results and Discussion

### 3.1. Phylogenetic and Protein Sequence Analyses of MATE Proteins

**Figure 1:**
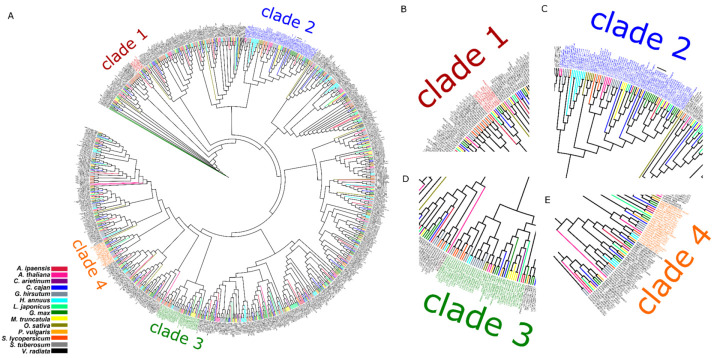
Phylogenetic analysis of the MATE family of proteins from 14 Leguminosae species (*Arachis ipaensis*, *Cajanus cajan*, *Cicer arietinum*, *Lotus japonicus*, *Medicago truncatula*, *Phaseolus vulgaris*, *Vigna radiata*, and *Glycine max*) and non-Leguminosae plants (*Arabidopsis thaliana*, *Gossypium hirsutum*, *Helianthus annuus*, *Oryza sativa*, *Solanum lycopersicum,* and *Solanum tuberosum*). The cluster consisting solely of MATE proteins from *Glycine max* is indicated by a black bar in clade 2. (**A**) The whole phylogenetic tree; (**B**) the enlarged portion of the phylogenetic tree showing clade 1; (**C**) the enlarged portion of the phylogenetic tree showing clade 2; (**D**) the enlarged portion of the phylogenetic tress showing clade 3; (**E**) the enlarged portion of the phylogenetic tree showing clade 4.

In order to gain insights into the structures of MATE proteins, we performed a phylogenetic analysis of MATE proteins from 14 representative plant species including some in the Leguminosae group (*Arachis ipaensis, Cajanus cajan, Cicer arietinum, Lotus japonicus, Medicago truncatula, Phaseolus vulgaris, Vigna radiata,* and *Glycine max*) and others in the non-Leguminosae group (*Arabidopsis thaliana, Gossypium hirsutum, Helianthus annuus, Oryza sativa, Solanum lycopersicum,* and *Solanum tuberosum*) which have a Benchmarking sets of Universal Single-Copy Orthologs (BUSCO) index [28] of their genomes higher than 85 (Figure 1). The results show that some clades (clades 1–4) are enriched with MATE proteins from Leguminosae (Figure 1). Within clade 2, there is a cluster consisting solely of MATE proteins from *Glycine max* (Glyma.03G005200, Glyma.03G005400, Glyma.19G120900, Glyma.03G005300, and Glyma.08G244400). This cluster is indicated by a black bar in clade 2 in Figure 1.

### 3.2. Identification of the Poly-Glutamate Motif in GmMATE4 by Sequence Alignment

**Figure 2:**
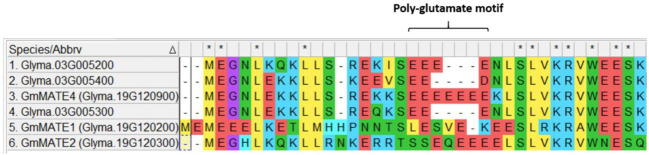
Alignment of the N-terminal amino acid sequences of Glyma.03G005200, Glyma.03G005300, Glyma.03G005400, GmMATE4, GmMATE1, and GmMATE2. The alignment of the full protein sequences is shown in Figure S2. The sequence alignment was done by ClustalW using MEGA11 [20].

In the cluster within clade 2 that consists solely of proteins from *G. max*, besides Glyma.08G244400 being predicted to be a truncated protein (Figure S1), Glyma.03G005200, Glyma.03G005300, and Glyma.03G005400 were all predicted to have 12 transmembrane domains (Figure S1). In this cluster, *Glyma.19G120900* has been previously identified to encode a MATE-type protein and was located in the overlapping QTLs regulating the contents of antioxidants, phenolics, and flavonoids in soybean seeds [17]. By aligning the protein sequences of these MATE proteins, a conserved poly-glutamate motif with a varied number of glutamate residues was found in the N-terminus (Figure 2). As a comparison, GmMATE1 and GmMATE2, which have also been reported to be located in the same overlapping QTLs with GmMATE4 [17], were also included in the protein sequence alignment. Among these MATE proteins, GmMATE4 has the most (seven consecutive) glutamate residues. Although Glyma.03G005400 has only two glutamate residues in the motif, it also has an aspartate residue, which is an acidic amino acid as well.

### 3.3. The Effect of the Poly-Glutamate Motif on the Protein Net Charge

Next, we investigated the functional significance of this poly-glutamate motif. Based on the topology prediction (Figure S2) and using Prot-pi [22], the N-termini of the MATE proteins (before the first transmembrane domain) were subjected to net charge prediction (Table 1). Since the close homologs, GmMATE1 and GmMATE2, were previously characterized to be localized at the vacuolar membrane [7], the net charge prediction was done at pH 5 or pH 5.5, which is the vacuolar pH [29] (Table 1). To assess the significance of the long string of consecutive glutamate residues in GmMATE4, mutations were created to produce a fragment in which three of the glutamate residues were substituted by alanine (GmMATE4Δ3ala) and a fragment in which all the glutamate residues were substituted by alanine (GmMATE4Δ7ala). The results showed that, among the MATE proteins in the cluster, the vacuolar domain of GmMATE4 was the most negatively charged at pH 5 or pH 5.5 (Table 1). Furthermore, the substitutions of glutamate residues by alanine (GmMATE4Δ3ala and GmMATE4Δ7ala) rendered the N-termini positively charged (Table 1). Next, we wanted to find out whether GmMATE4 is an isoflavone transporter, like its homolog GmMATE1 and GmMATE2, and whether the alanine substitutions of this poly-glutamate motif affect the function of GmMATE4.

### 3.4. Subcellular Localization Study

**Figure 3:**
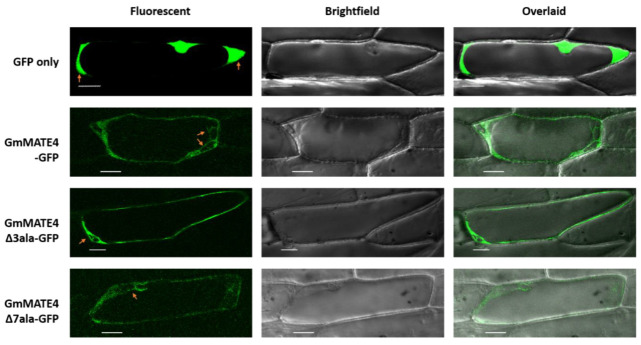
Subcellular localization study of GmMATE4, GmMATE4Δ3ala, and GmMATE4Δ7ala. GFP was fused to the C-termini of GmMATE4, GmMATE4Δ3ala, and GmMATE4Δ7ala. The fusion construct was cloned downstream of a CaMV 35S promoter in the plasmid V7. The plasmid was coated onto gold particles, bombarded into onion epidermal cells, and then, observed using a confocal microscope. Scale bar, 50 μm; excitation 488 nm; and the emission signal was collected between 500 and 545 nm. All cells having the green, fluorescent signal (≥11 cells) from two biological repeats showed the same patterns.

The close homologs of GmMATE4, GmMATE1, and GmMATE2 were reported to be localized in the vacuolar membrane [7]. As a confirmation of the subcellular localization of GmMATE4, GmMATE4Δ3ala, and GmMATE4Δ7ala, the genes encoding these proteins were fused with *green fluorescent protein* (*GFP*). The fusion constructs, *GmMATE4-GFP*, *GmMATE4Δ3ala-GFP*, or *GmMATE4Δ7ala-GFP* were coated on gold particles and bombarded into onion epidermal cells. The green fluorescence signal was, then, detected using a confocal microscope. The results showed that GmMATE4-GFP, GmMATE4Δ3ala-GFP, and GmMATE4Δ7ala-GFP were preferably localized in the vacuolar membrane (Figure 3). Based on the topology prediction and the subcellular localization study, the N-termini used for the net charge prediction of GmMATE4, GmMATE4Δ3ala, and GmMATE4Δ7ala (Table 1) were confirmed to be a vacuolar domain.

### 3.5. The Transporter Activity of GmMATE4

**Figure 4:**
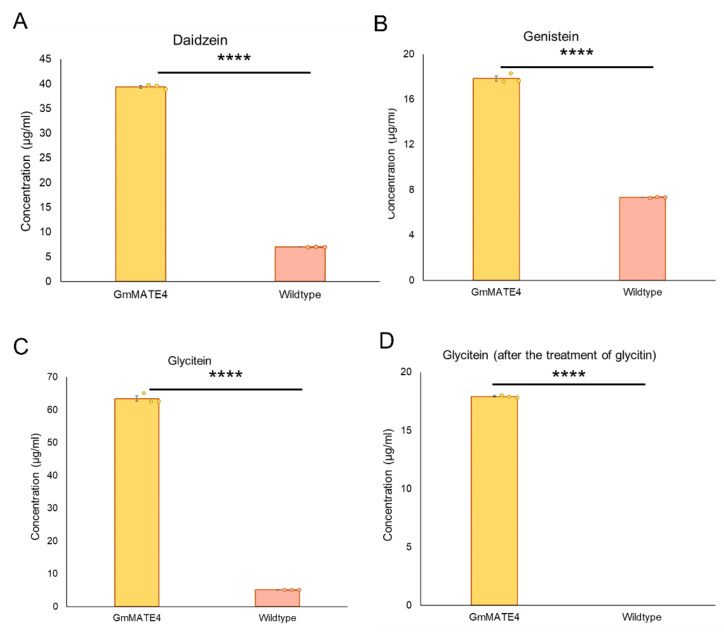
Uptake assays of isoflavones. The isoflavones detected in the yeast cells after the treatments of: (**A**) daidzein; (**B**) genistein; (**C**) glycitein; or (**D**) glycitin. Error bar: standard error from three technical repeats. Student’s *t* test (two-tailed) was performed to compare the isoflavone uptake capacity between the constructs. **** indicates *p* < 0.0001. The experiments were performed twice with similar results.

The close homologs of GmMATE4, GmMATE1, and GmMATE2 were reported to be isoflavone transporters [7]. To test whether GmMATE4 is also an isoflavone transporter, using yeast as the model, *GmMATE4* was ectopically expressed in yeast cells. Untransformed yeast cells (wildtype) and *GmMATE4* expressing yeast cells were subjected to isoflavone uptake assays. The yeast cells were treated with individual isoflavones, including aglycones (daidzein, genistein, and glycitein), isoflavone glucosides (daidzin, genistin, and glycitin), and malonylated (M-) isoflavones (M-daidzin, M-genistin, and M-glycitin). The results of the uptake assays showed that, as compared with the wildtype, *GmMATE4* expressing yeast cells had significantly higher amounts of daidzein (Figure 4A), genistein (Figure 4B), and glycitein (Figure 4C) after the respective isoflavone treatments. When the yeast cells were treated with glycitin, a significantly higher amount of glycitein was found in *GmMATE4* expressing yeast cells as compared with the wildtype (Figure 4D). The chromatograms showing the detection of isoflavones after all the treatments are shown in Supplementary Figures S4–S6. The chromatograms of the mock treatment, in which only DMSO but not any isoflavone was added to the yeast cells, are shown in Supplementary Figure S7.

### 3.6. The Functional Significance of the Poly-Glutamate Motif on the Transporter Activity of GmMATE4

**Figure 5:**
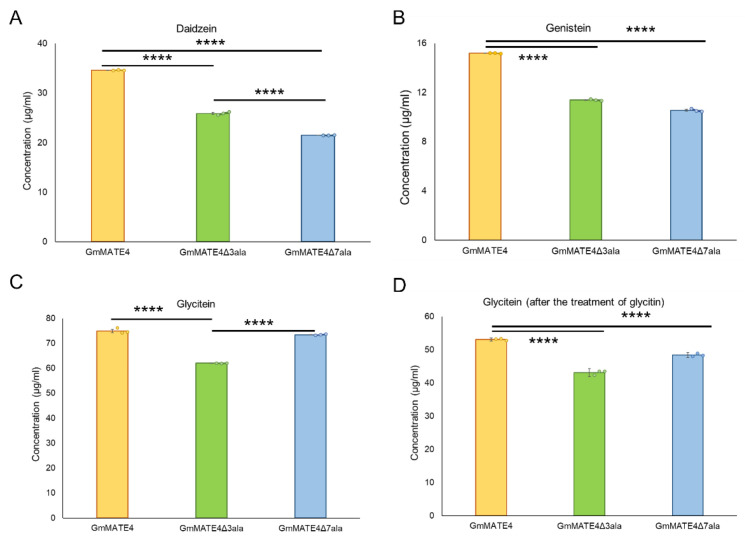
Uptake assays of isoflavones. The isoflavones detected in the yeast cells after the treatments of (**A**) daidzein, (**B**) genistein, (**C**) glycitein, and (**D**) glycitin. Error bar: standard error from three technical repeats. Student’s *t* test (two-tailed) was performed to compare the isoflavone uptake capacity between the constructs. **** indicates *p* < 0.0001. The experiments were performed twice with similar results.

The above results suggested that GmMATE4 mediates the uptake of daidzein, genistein, glycitein, and glycitin (Figure 4). Then, we tested the functional significance of the poly-glutamate motif on the activity of GmMATE4 to transport these isoflavone species (daidzein, genistein, glycitein, and glycitin). *GmMATE4*, *GmMATE4Δ3ala*, or *GmMATE4Δ7ala* was ectopically expressed in yeast cells for isoflavone uptake assays. The yeast cells were treated with daidzein, genistein, glycitein, or glycitin. The results of the uptake assays showed that, as compared with GmMATE4, GmMATE4Δ3ala and GmMATE4Δ7ala had weakened capacities to mediate the transport of daidzein (Figure 5A) and genistein (Figure 5B). GmMATE4Δ3ala and GmMATE4Δ7ala had similar capacities to mediate the transport of genistein (Figure 5B) but GmMATE4Δ7ala had even weaker capacity to mediate the transport of daidzein as compared with GmMATE4Δ3ala (Figure 5A). As compared with GmMATE4, GmMATE4Δ3ala had weakened capacity to transport glycitein (Figure 5C). However, as compared with GmMATE4, GmMATE4Δ7ala had similar capacity transport glycitein (Figure 5C). When the cells were treated with glycitin, a significantly higher amount of glycitein was found in both *GmMATE4Δ3ala* expressing yeast cells and *GmMATE4Δ7ala* expressing yeast cells as compared with *GmMATE4* expressing yeast cells (Figure 5D). The chromatograms showing the detection of isoflavones after the treatment of daidzein, genistein, glycitein, and glycitin in *GmMATE4*, *GmMATE4Δ3ala*, or *GmMATE4Δ7ala* expressing yeast cells are shown in Supplementary Figure S8.

## 4. Discussion

Structural analyses have been done to understand the functional significance of each domain of MATE transporters. However, previous studies have largely been biased towards understanding the TMDs rather than the N- and C-termini [1,2,3]. In addition, studies on other transporter proteins have revealed that the N- and C-termini could also play important regulatory roles [15].

To improve the understanding of the protein domains of MATE transporters in plants, MATE transporters from 14 representative plant species, including Leguminosae and non-Leguminosae, were subjected to phylogenetic analyses (Figure 1). Among the clades enriched with MATE proteins from Leguminosae, there was a cluster consisting solely of MATE proteins from *G. max* (Figure 1). Protein sequence alignment of members in this cluster showed a motif rich in glutamate residues (Figure 2). This motif is, thus, named a poly-glutamate motif. The substitution of the negatively charged glutamate residues by alanine reduced the overall negative charge of this domain as expected (Table 1). Glutamate residues have been suggested to be involved in proton transport. For example, the D35, D38, and D144 in the TMDs of AtPHT1;1 (*Arabidopsis thaliana* phosphate transporter 1;1) have been reported to play an essential role in proton transfer by forming a conduit that facilitates proton transport across membranes [30]. The mutation of any of these residues to alanine hampered the phosphate transport activity [30]. In addition, the glutamate residue, named gating glutamate (Glu_gate_) and conserved in the CLC (chloride channels) family of proteins, has been suggested to serve as a gate for proton transit in CLC antiporters [31,32,33,34]. The mutation of this glutamate residue to alanine (E148A) in EcCLC (*Escherichia coli* CLC) hampered proton transport as compared with the wild type [34]. The mutation of threonine to glutamate (T269E) in CmCLC (CLC from red algae) promoted proton transport as compared with the wild type [34]. The above examples show the importance of glutamate residues in TMDs for promoting proton transport. Despite these findings, the importance of glutamate residues in the N- or C-terminus, and the functional significance of consecutive glutamate residues, remain to be unclear. Nevertheless, based on the above examples, it could be deduced that glutamate residues play an important role in promoting proton affinity. In addition, the attraction of proton by the production of polyglutamic acid on the cell surface has been reported in *Bacillus pseudofirmus* OF4 cells [35,36]. It was suggested that such attraction of protons to the cell surface was associated with the formation of proton motive force across the membrane [36]. It is possible that the polyglutamate motif of GmMATE4 plays a similar role in attracting protons to the vacuolar membrane surface. Previous studies have shown that MATE transporters in eukaryotes are antiporters that transport their substrates in exchange for H^+^ [4]. The proton gradient across the membrane drives the transport of the substrate [4]. Therefore, it is logical to deduce that the poly-glutamate motif of GmMATE4 at the vacuolar domain is associated with the attraction of protons to the membrane surface, and thus, promote the proton motive force and the transport of the substrates into the vacuole.

The results of the isoflavone uptake assays were in line with the above deduction. Similar to GmMATE1 and GmMATE2, which are close homologs of GmMATE4, GmMATE4 could mediate the transport of isoflavones including daidzein, genistein, glycitein, and glycitin (Figure 4). The mutation of glutamate residues to alanine (GmMATE4Δ3ala or GmMATE4Δ7ala) reduced the isoflavone transport activity of the mutant proteins as compared with the native GmMATE4 (Figure 5). Among the substrates being transported, GmMATE4Δ3ala or GmMATE4Δ7ala showed differential capacity to transport daidzein, with GmMATE4Δ3ala having a higher transport activity as compared with GmMATE4Δ7ala (Figure 5A). It is possible that a further reduction in the net charge of the N-terminus of GmMATE4Δ7ala (Table 1) led to a further reduction in the transport activity. However, such phenomenon was not observed when the cells were treated with genistein (Figure 5B) or glycitein (Figure 5C). In addition, when the cells were treated with glycitein, GmMATE4Δ3ala had reduced transport capacity as compared with the native GmMATE4, as expected (Figure 5C), while GmMATE4Δ7ala and the native GmMATE4 had a similar capacity to transport glycitein (Figure 5C). Such observations are possibly due to the different mechanics of the transport of different substrates and the potential effects of the mutation on the interaction with other protein motifs. A detailed mechanistic study would be needed to delineate the regulation.

Similar to GmMATE1 and GmMATE2 [7], which are the close homologs of GmMATE4, in this study, we showed that GmMATE4 localizes at the vacuolar membrane-like structure (Figure 3). Together with the isoflavone uptake assays (Figure 4), the results indicated that GmMATE4 has similar functions as GmMATE1 and GmMATE2, which were previously demonstrated to be involved in the storage of isoflavones in the vacuole. However, the longer consecutive glutamate residues at the vacuolar domain of GmMATE4 as compared with GmMATE1 and GmMATE2 (Figure 2) possibly allows further regulation of the transporter function.

## 5. Conclusions

In this study, by the phylogenetic analysis of MATE proteins from 14 plant species, we identified a cluster consisting of four full-length MATE proteins in soybean. By aligning the four full-length MATE proteins, a poly-glutamate motif was identified. Among the four MATE proteins, GmMATE4 has the most consecutive glutamate residues at the N-terminus, which is the vacuolar domain. Net charge prediction suggested the importance of the poly-glutamate motif to the negative charge of the N-termini of GmMATE4. In this study, GmMATE4 was shown to be localized at the vacuolar membrane-like structure. GmMATE4 was also found to mediate the uptake of isoflavones including daidzein, genistein, glycitein, and glycitin. The mutagenesis and isoflavone uptake assays show that the poly-glutamate motif of GmMATE4 regulates its activity to transport daidzein, genistein, glycitein, and glycitin. To the best of our knowledge, this is the first study reporting the isoflavone transport activity of GmMATE4 and the functional significance of poly-glutamate motif on the transporter activity of MATE protein.

## Figures and Tables

**Table 1:** Net charge prediction of the N-termini of MATE-type proteins.

	Net Charge of the Vacuolar N-Terminal Region		% of Acidic Amino Acids (No. of Acidic Amino Acids/Total Amino Acids)
	pH 5	pH 5.5	
Glyma.03G005200	+0.773	−0.37	24.2% (8/33)
Glyma.03G005400	−1.881	−3.245	29.4% (10/34)
Glyma.03G005300	+0.794	−0.363	23.5% (8/34)
GmMATE1 (Glyma.19G120200)	+0.3	−1.534	27.5% (11/40)
GmMATE2 (Glyma.19G120300)	+2.708	+1.426	21.6% (8/37)
GmMATE4 (Glyma.19G120900)	−0.348	−2.043	33.3% (12/36)
GmMATE4Δ3ala	+1.992	+0.712	25% (9/36)
GmMATE4Δ7ala	+5.113	+4.385	13.9% (5/36)

## Data Availability

All data generated in this study are available within this manuscript and companion Supplementary Materials.

## Supplementary Materials

The following supporting information can be downloaded at https://www.mdpi.com/article/10.3390/membranes12020206/s1. Figure S1: The predicted transmembrane topologies of GmMATE transporters, Figure S2: Alignment of the full protein sequences of Glyam.03G005200, Glyma.03G005300, Glyma.03G005400, GmMATE4 (Glyma.19G120900), GmMATE1 (Glyma.19G120200), and GmMATE2 (Glyma.19G120300), Figure S3: A UHPLC chromatogram showing the absorption peaks of the nine isoflavone standards, Figure S4: Representative UHPLC chromatograms of the detection of isoflavones in the wildtype and *GmMATE4* expressing yeast cells after the treatment with daidzein, genistein, and glycitein, Figure S5: Representative UHPLC chromatograms of the detection of isoflavones in the wildtype and *GmMATE4* expressing yeast cells after the treatment with daidzin, genistin, and glycitin, Figure S6: Representative UHPLC chromatograms of the detection of isoflavones in the wildtype and *GmMATE4* expressing yeast cells after the treatment with malonyl-daidzin, malonyl-genistin, and malonyl-glycitin, Figure S7: Representative UHPLC chromatograms of the detection of isoflavones after the mock treatment, Figure S8: Representative UHPLC chromatograms of the detection of isoflavones in GmMATE4, GmMATE4Δ3ala, or GmMATE4Δ7ala expressing yeast cells after the treatment with daidzein, genistein, glycitein, and glycitin, Table S1: List of primers and restriction enzymes used for cloning.
