# Supplementary material for: The Poly-Glutamate Motif of GmMATE4 Regulates Its Isoflavone Transport Activity
Source: Membranes (Basel). 2022 Feb 10;12(2):206. doi: 10.3390/membranes12020206 (PMC8880658; doi:10.3390/membranes12020206)
Supplement: Supplementary file 1 [file membranes-12-00206-s001.zip › membranes-1563222-supplementary.pdf]

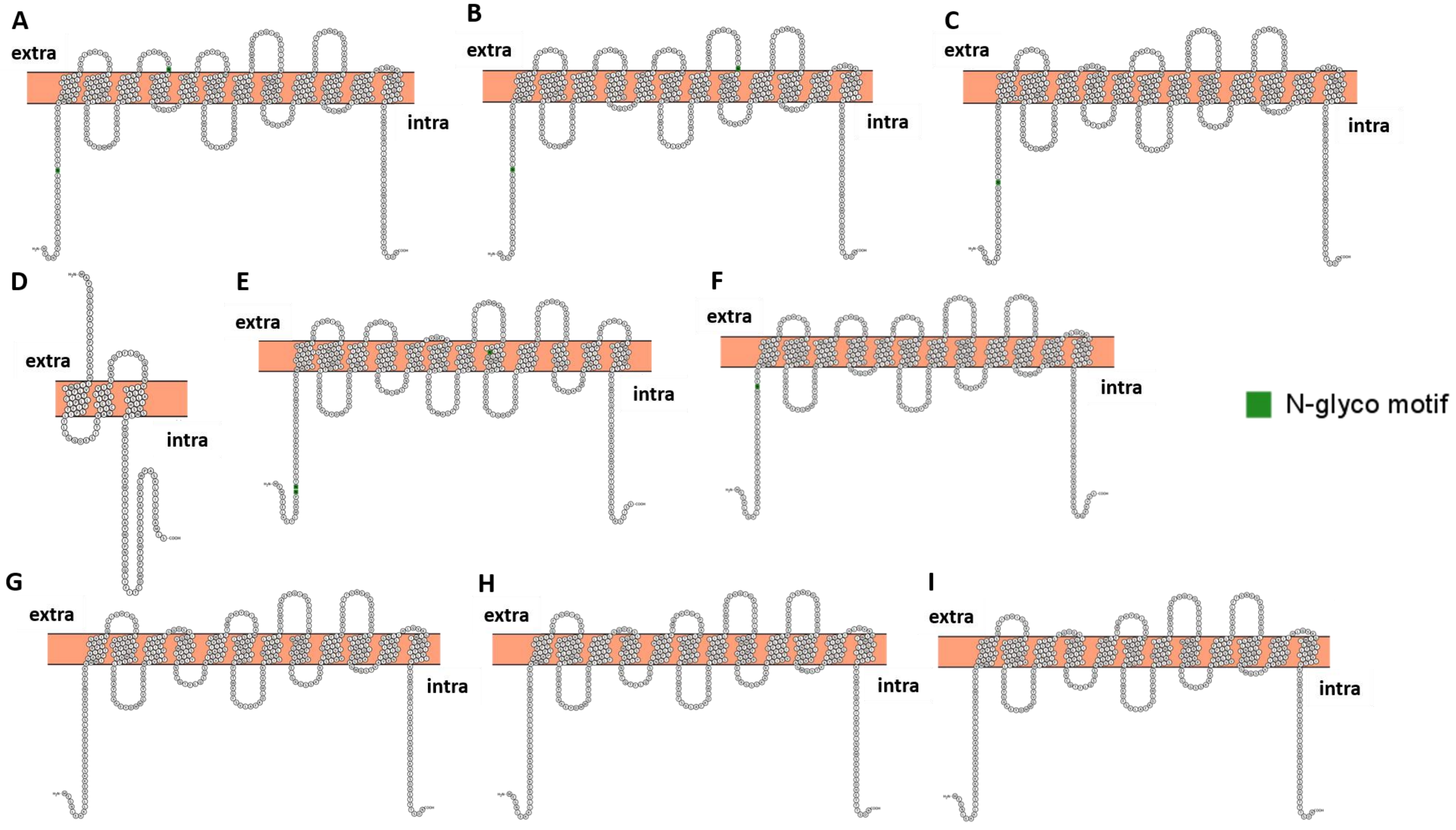

Figure S1. The predicted transmembrane topologies of GmMATE transporters. **A**. Glyma.03G005200, **B**. Glyma.03G005300, **C**. Glyma.03G005400, **D**. Glyma.08G244400, **E**. GmMATE1 (Glyma.19G120200), **F**. GmMATE2 (Glyma.19G120300), **G**. GmMATE4 (Glyma.19G120900), **H**. GmMATE4Δ3ala, and **I**. GmMATE4Δ7ala. The transmembrane topologies were predicted using Protter [1].

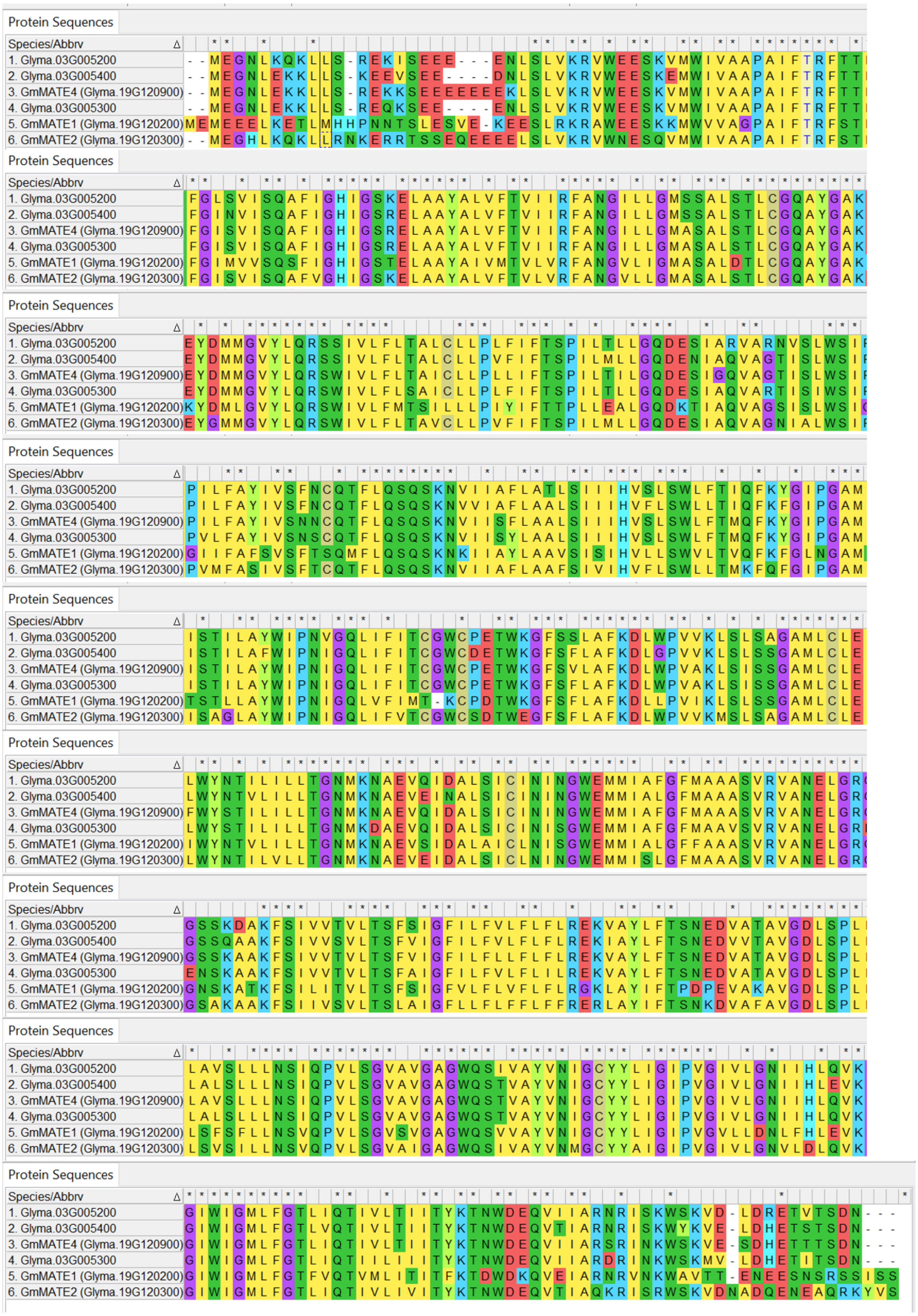

Figure S2. Alignment of the full protein sequences of Glyma.03G005200, Glyma.03G005300, Glyma.03G005400, GmMATE4 (Glyma.19G120900), GmMATE1 (Glyma.19G120200), and GmMATE2 (Glyma.19G120300). The sequence alignment was done by ClustalW using MEGA11 [2].

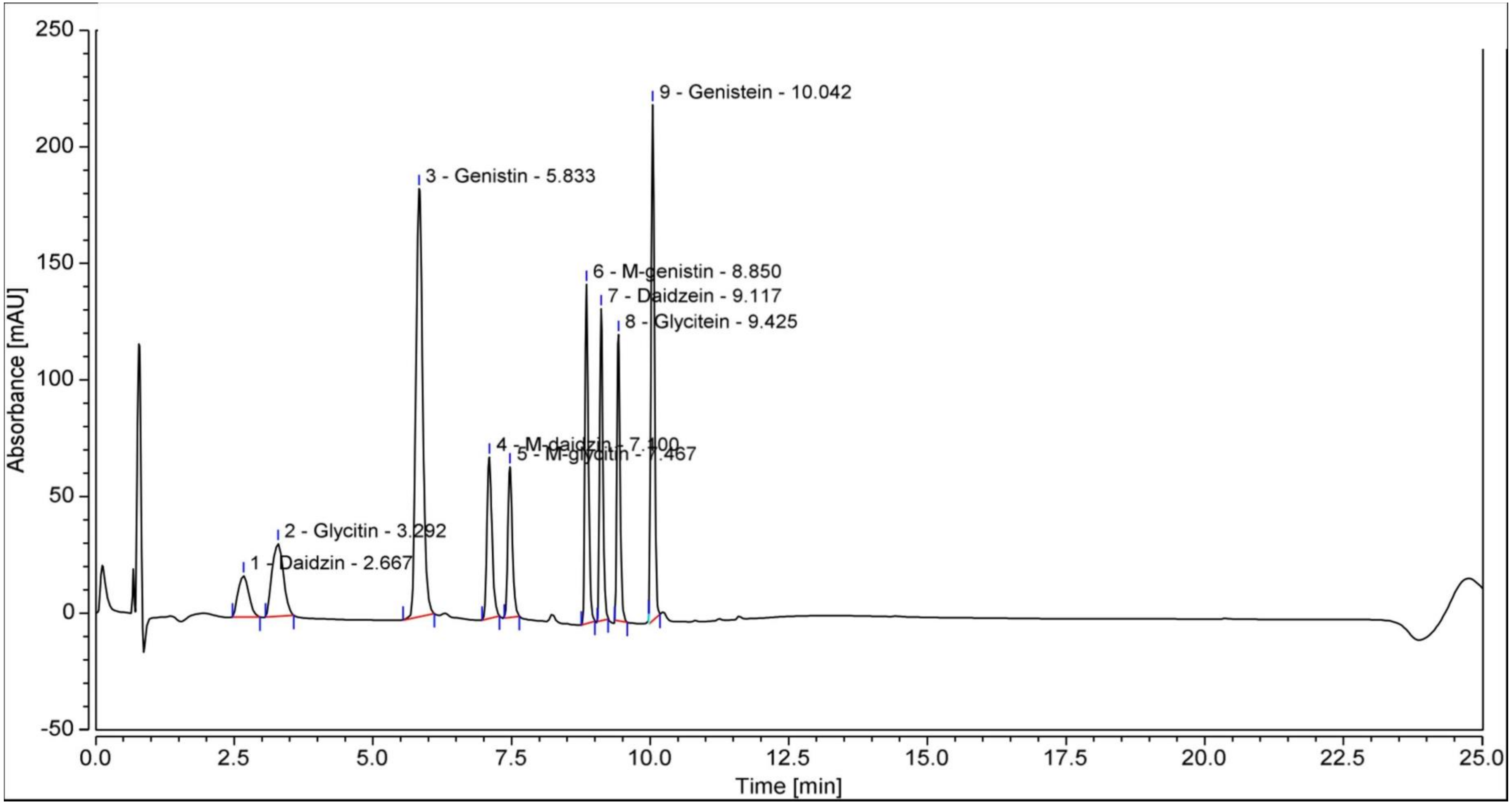

Figure S3. A UHPLC chromatogram showing the absorption peaks of the nine isoflavone standards. One microliter of a mixture of the isoflavones at 20ppm each was injected into the UHPLC system. Peak 1: Daidzin; Peak 2: Glycitin; Peak 3: Genistin; Peak 4: Malonyl-daidzin; Peak 5: Malonyl-glycitin; Peak 6: Malonyl-genistin; Peak 7: Daidzein; Peak 8: Glycitein; Peak 9: Genistein.

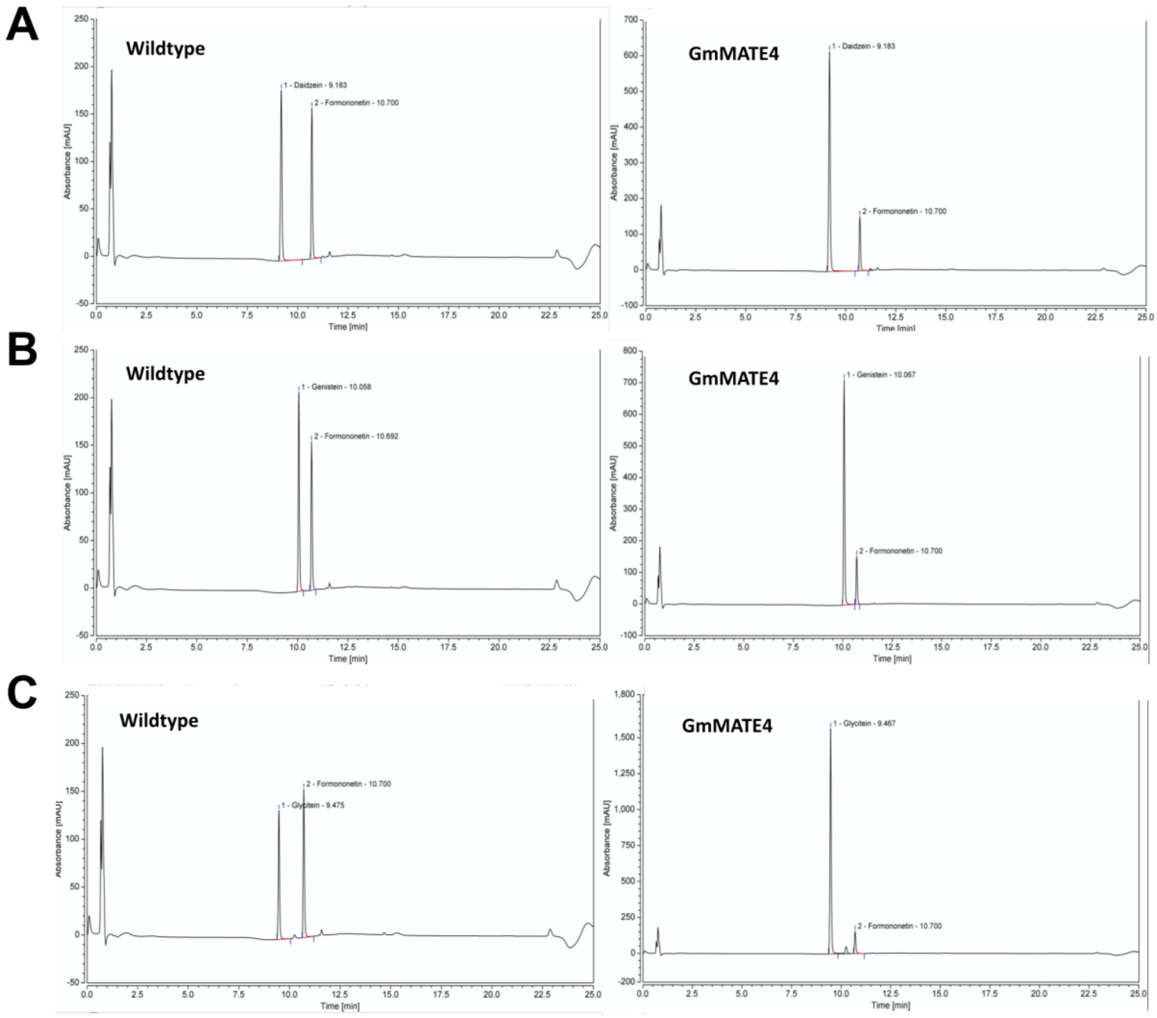

Figure S4. Representative UHPLC chromatograms of the detection of isoflavones in the wildtype and *GmMATE4* expressing yeast cells after the treatment with **(A)** daidzein, **(B)** genistein, and **(C)** glycitein.

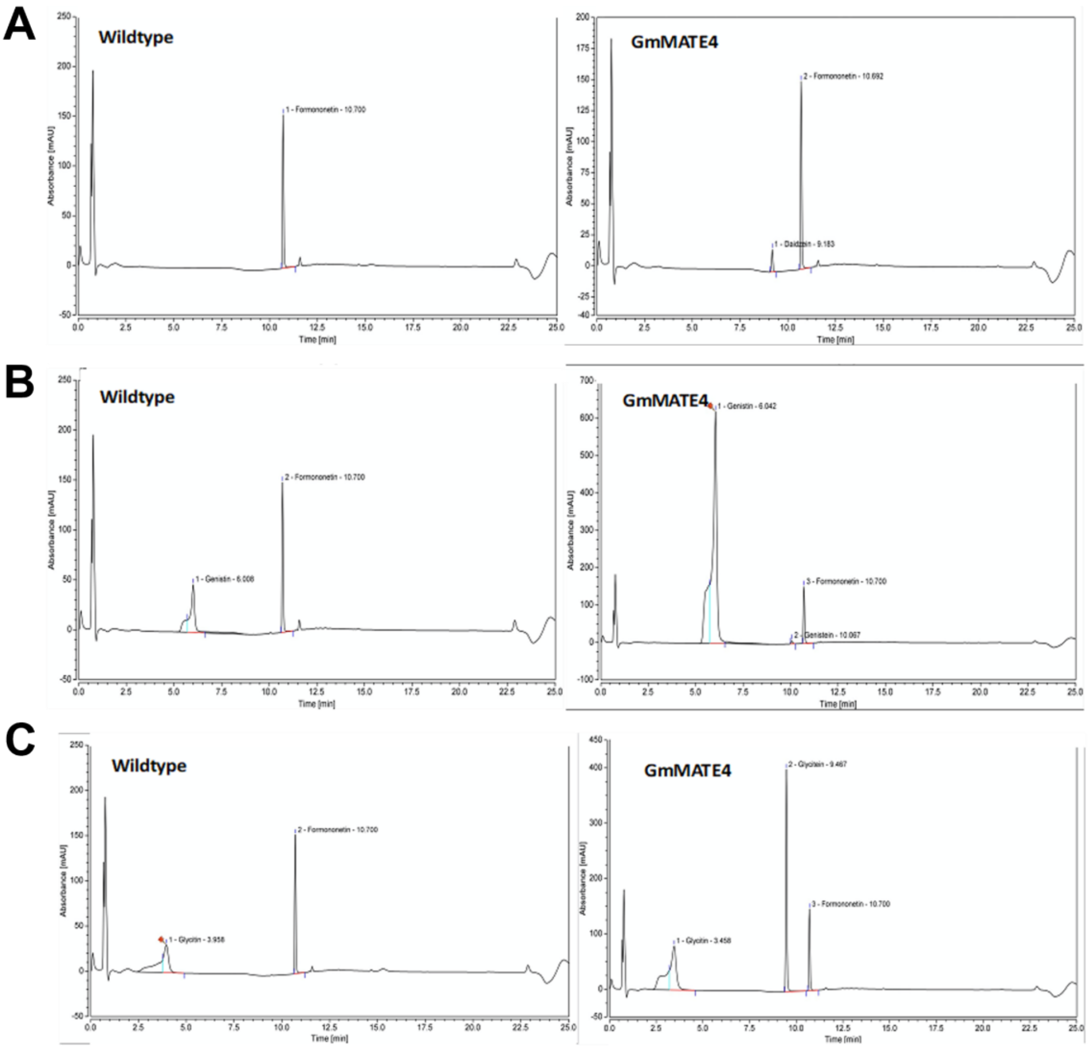

Figure S5. Representative UHPLC chromatograms of the detection of isoflavones in the wildtype and *GmMATE4* expressing yeast cells after the treatment with **(A)** daidzin, **(B)** genistin, and **(C)** glycitin.

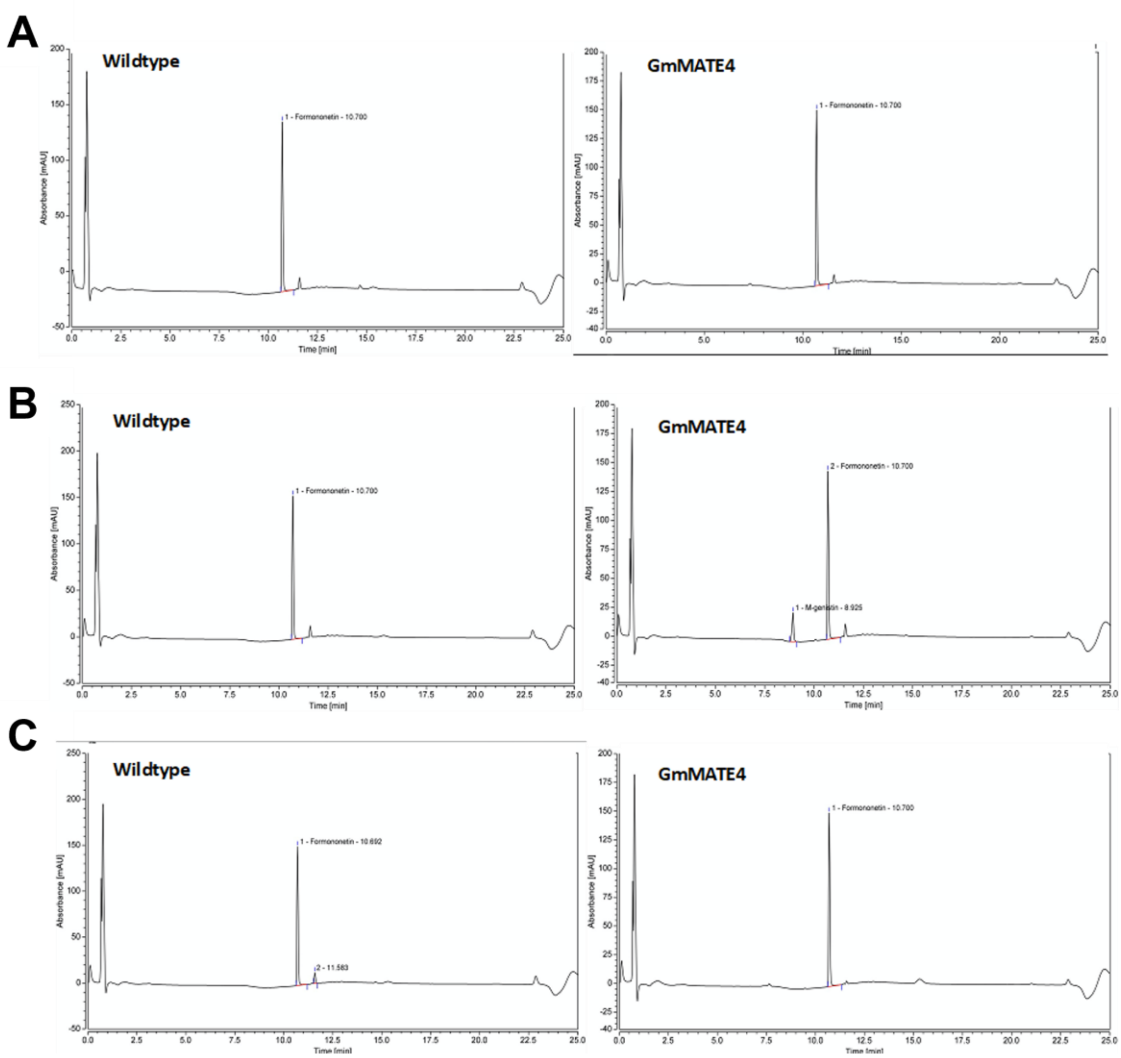

Figure S6. Representative UHPLC chromatograms of the detection of isoflavones after the treatment with **(A)** malonyl-daidzin, **(B)** malonyl-genistin, **(C)** and malonyl-glycitin.

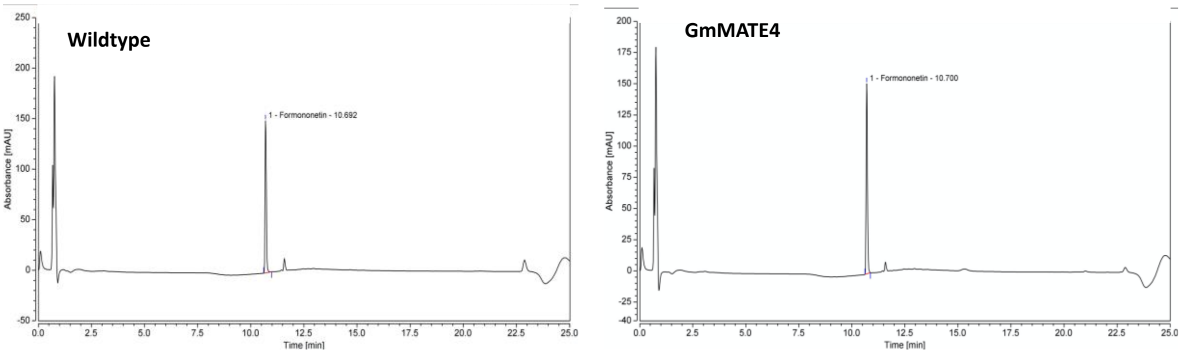

Figure S7. Representative UHPLC chromatograms of the detection of isoflavones after the mock treatment.

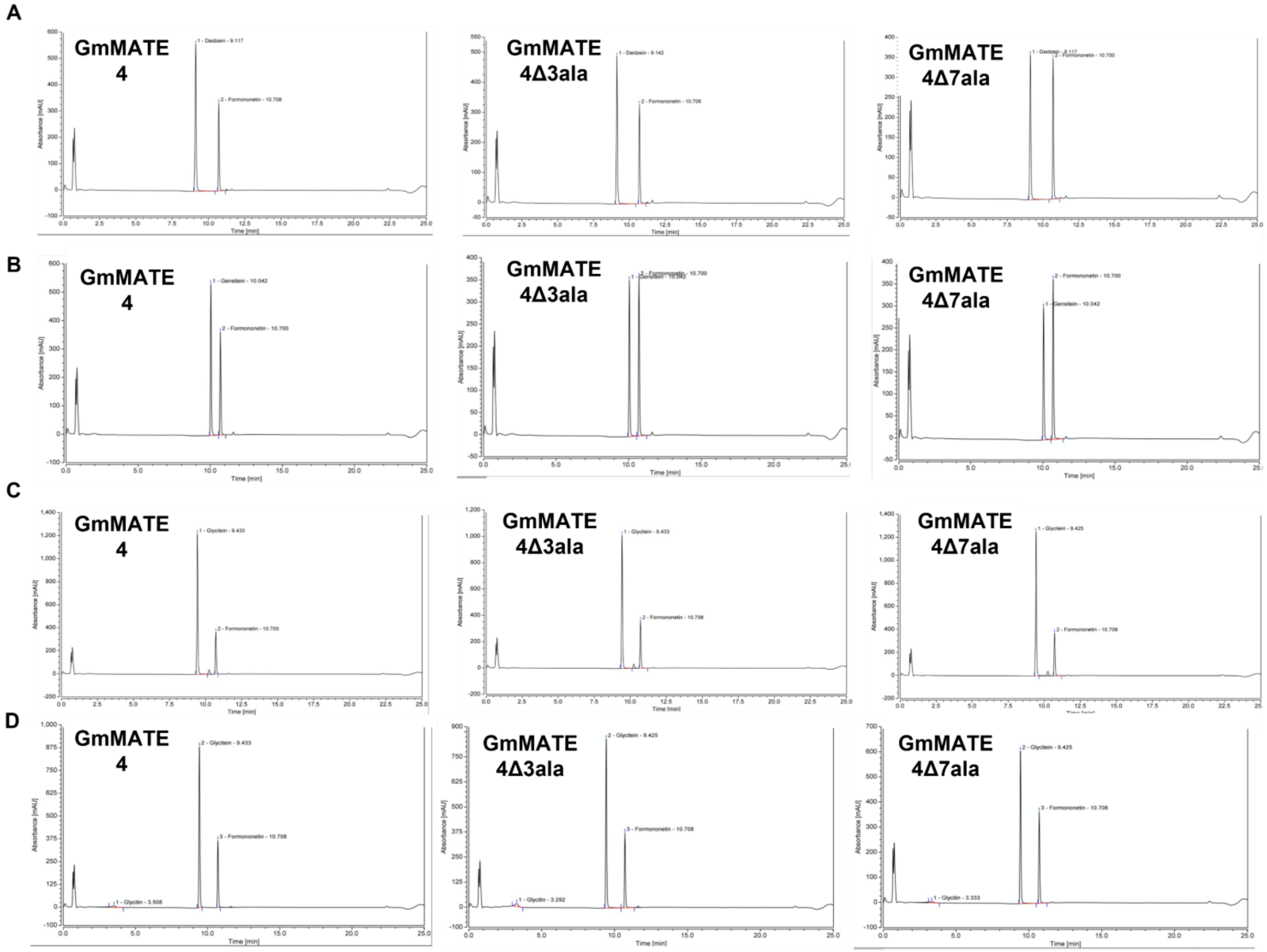

Figure S8. Representative UHPLC chromatograms of the detection of isoflavones in *GmMATE4*, *GmMATE4Δ3ala,* or *GmMATE4Δ7ala* expressing yeast cells after the treatment with **(A)** daidzein, **(B)** genistein, **(C)** glycitein, and **(D)** glycitin.

Table S1. List of primers and restriction enzymes used for cloning.

**Primers and restriction enzymes**	**Primer sequences (5' - 3')**
Cloning of *GmMATE4* from into pGBKT7ΔBD, forward, SmaI	ACCCGGGAATGGAGGGGAATCTAG
Cloning of *GmMATE4* from into pGBKT7ΔBD, reverse, SalI	AAAGTCGACCTAATTATCTGATGTAGTTG
Cloning of *GmMATE4Δ3ala* into pGBKT7ΔBD, forward	AATGGAGGGGAATCTAGAGAAGAAGCTGTTGAGCAGAGAAAAAAAATCAGCTGCTGCTGAAGAAGAAGAGAAA
Cloning of *GmMATE4Δ3ala and GmMATE4Δ7ala* into pGBKT7ΔBD, reverse, SalI	AAGTCGACCTAATTATCTGATGTAGTTGTTTCATGATCAGATTCCAC
Cloning of *GmMATE4Δ7ala* into pGBKT7ΔBD, forward	AATGGAGGGGAATCTAGAGAAGAAGCTGTTGAGCAGAGAAAAAAAATCAGCTGCTGCTGCTGCTGCTGCTAAATTATCAT
Cloning of *GmMATE4*, *GmMATE4Δ3ala* and *GmMATE4Δ7ala* for fusing with *GFP* in V7, forward, NheI	AAAAAGCTAGCATGGAGGGGAATCTAG
Cloning of *GmMATE4*, *GmMATE4Δ3ala* and *GmMATE4Δ7ala* for fusing with *GFP* in V7, reverse, XhoI	AAAAACTCGAGATTATCTGATGTAGTTG

References

1. Omasits, U.; Ahrens, C.H.; Müller, S.; Wollscheid, B. Protter: Interactive protein feature visualization and integration with experimental proteomic data. *Bioinformatics* **2014**, *30*, 884–886.
2. Tamura, K.; Stecher, G.; Kumar, S. MEGA11: Molecular evolutionary genetics analysis version 11. *Mol. Biol. Evol.* **2021**, *38*, 3022–3027.
